# *Neoshirakia japonica* (Siebold & Zucc.) Esser [Euphorbiaceae] fruit suppresses obesity and obesity-induced inflammation in adipocytes, macrophages, and obese mice

**DOI:** 10.3389/fphar.2025.1647343

**Published:** 2025-09-29

**Authors:** Eunbi Lee, Juhye Park, Ju-Ock Nam

**Affiliations:** ^1^ Department of Food Science and Biotechnology, Kyungpook National University, Daegu, Republic of Korea; ^2^ Department of Integrative Biology, Kyungpook National University, Daegu, Republic of Korea; ^3^ Research Institute of Tailored Food Technology, Kyungpook National University, Daegu, Republic of Korea; ^4^ Department of Advanced Bioconvergence, Kyungpook National University, Daegu, Republic of Korea

**Keywords:** Neoshirakia japonica (Siebold & Zucc.) Esser [Euphorbiaceae], obesity, inflammation, adipogenesis, phytotherapy

## Abstract

**Introduction:**

The objective of this study was to demonstrate the anti-obesity and anti-inflammatory effects of *Neoshirakia japonica* (Siebold & Zucc.) Esser [Euphorbiaceae] under obesity-induced conditions. Traditionally used in ethnopharmacology to dispel “wind-dampness” and “damp-heat,” these effects can be interpreted in modern medicine as chronic inflammation and metabolic imbalance associated with obesity.

**Methods:**

The anti-obesity and anti-inflammatory potential of *N. japonica* fruit extract (NJFE) was evaluated in high-fat diet (HFD)-induced obese mice, 3T3-L1 adipocytes, and LPS-stimulated RAW264.7 macrophages. Mitochondrial function was assessed using MitoTracker fluorescence, and LC-MS analysis was performed to identify the chemical fingerprint and putative bioactive metabolites.

**Results:**

In HFD-induced obese mice, NJFE significantly reduced adipocyte hypertrophy, dyslipidemia, and glucose intolerance, while alleviating adipose tissue inflammation. In 3T3-L1 adipocytes, NJFE suppressed adipogenic differentiation and downregulated adipogenesis-related genes. In RAW264.7 macrophages stimulated with LPS, NJFE markedly reduced inflammatory responses. Furthermore, NJFE attenuated inflammatory responses in 3T3-L1 adipocytes exposed to conditioned medium derived from LPS-stimulated RAW264.7 cells. NJFE also improved mitochondrial function in adipocytes.

**Conclusion:**

These findings demonstrate that NJFE not only improves obesity but also alleviates obesity-induced inflammation, supporting its potential as a promising natural therapeutic candidate for the prevention and treatment of obesity and related metabolic disorders.

## 1 Introduction

Obesity, independently or in conjunction with other diseases, induces many metabolic disorders (Kopelman, 2000). According to life insurance data and epidemiological studies, as the degree to which a person is overweight or obese increases, there is a significant increase in mortality after 15 years (Lew, 1985). In the United States, the total economic cost of obesity is estimated to be around $99.2 billion, with direct medical costs related to obesity accounting for approximately 5.7% of total healthcare expenditures, highlighting obesity’s significant impact not only on health but also on society (Am, 1998). Additionally, body fat and obesity are closely related to aesthetics, an aspect that has consistently attracted attention. Obesity significantly impacts social perceptions related to appearance, which can, in turn, affect an individual’s self-esteem and social relationships. Therefore, the effects of excess body fat and obesity are addressed as not only health concerns but also social and cultural issues (Wang et al., 2004).

In addition to its effects on appearance, excessive body fat triggers inflammation within the body. The inflammatory response associated with obesity begins when macrophages are recruited to adipose tissue, where they secrete inflammatory cytokines, such as TNFα, IL-6, and MCP1 These inflammatory cytokines activate the NF-κB signaling pathway within the adipose tissue (Shoelson et al., 2007), which inhibits insulin signaling and its downstream signaling pathways, resulting in insulin resistance and glucose intolerance (Wu and Ballantyne, 2020). Therefore, new treatments that can effectively reduce obesity and its complications are urgently needed. Existing conventional pharmaceutical-based obesity treatments have limitations, including various side effects and sustainability issues (Atkinson, 1997). Orlistat, for example, is a commonly used drug that inhibits the absorption of dietary fats by blocking their breakdown; however, its effectiveness tends to diminish over time during prolonged treatment. Another currently available anti-obesity agent is liraglutide, a glucagon-like peptide-1 (GLP-1) receptor agonist. Although GLP-1 exhibits appetite-suppressing effects, it is often associated with gastrointestinal intolerance and nausea. Given these limitations, there is growing interest in plant-derived natural products as alternative strategies for obesity management. Natural metabolites extracted from medicinal plants are generally considered to have fewer side effects, making them safer and more sustainable therapeutic options (Shaik Mohamed Sayed et al., 2023).

*Neoshirakia japonica* (Siebold & Zucc.) Esser [Euphorbiaceae] (syn. *Sapium japonicum*) is a deciduous shrub belonging to the Euphorbiaceae family (Ahn et al., 1996; Alam et al., 2020; Ohigashi et al., 1972). According to Zhong Yao Da Ci Dian, *Neoshirakia japonica* (Siebold & Zucc.) Esser [Euphorbiaceae] has been ethnopharmacologically used to dispel “*fengshi*” (wind-dampness) and eliminate “*shirer*” (damp-heat). In relation to this, modern medicine has reported that *Neoshirakia japonica* (Siebold & Zucc.) Esser [Euphorbiaceae] has beneficial effects on relieving overfatigue, low back pain and knee pain as well as possessing antioxidant properties (He et al., 2021; Lai et al., 2004). What is noteworthy here is that the traditional Chinese medicine concept of “dispelling wind-dampness” can be interpreted as having anti-inflammatory effects in modern terms (Ge et al., 2020), and “damp-heat” can be understood as a pathological condition contributing to inflammatory and obesity-related metabolic disorders (Dimitrakopoulou et al., 2022; Le Huërou-Luron et al., 2010; Zhao et al., 2023a). Therefore, the ethnopharmacological effects of *Neoshirakia japonica* (Siebold & Zucc.) Esser [Euphorbiaceae] in eliminating wind-dampness and damp-heat can be reinterpreted as potential therapeutic effects on obesity and metabolic syndrome in modern medicine.

Supporting this possibility, *Neoshirakia japonica* (Siebold & Zucc.) Esser [Euphorbiaceae] has been reported to contain Neojaponicin A, B, C, D and E (Zhao et al., 2023b). In addition, it contains kaempferol along with a variety of kaempferol derivatives, including kaempferol 3-O-(6″-O-feruloyl)-β-D-galactopyranoside, kaempferol 3-O-(2″-O-galloyl-6″-O-feruloyl)-β-D-galactopyranoside, kaempferol 3-O-(2″-O-galloyl-6″-O-p-coumaroyl)-β-D-galactopyranoside, kaempferol 3-O-(2″-O-galloyl-6″-O-p-coumaroyl)-β-D-glucopyranoside, and kaempferol 3-O-(2″-O-galloyl-6″-O-feruloyl)-β-D-glucopyranoside (Zhao et al., 2021).

This study presents *Neoshirakia japonica* (Siebold & Zucc.) Esser [Euphorbiaceae] fruit extract as a potential agent for the treatment of obesity and the inflammation and glucose intolerance associated with it. The anti-obesity and metabolic syndrome-improving effects of *Neoshirakia japonica* (Siebold & Zucc.) Esser [Euphorbiaceae] may be interpreted as stemming from its ethnopharmacologically reported actions of eliminating damp-heat and wind-dampness. However, to date, no studies have scientifically validated its efficacy against obesity in the context of modern medicine. Therefore, we propose *Neoshirakia japonica* (Siebold & Zucc.) Esser [Euphorbiaceae] as a novel candidate for targeting obesity and obesity-related metabolic syndrome. According to our research, *N. japonica* fruit extract (NJFE) not only reduces body fat but also inhibits the secretion of inflammatory cytokines and regulates the NF-κB signaling pathway, showing potential for use in the treatment of obesity-induced insulin resistance. This suggests a new approach to obesity treatment and is expected to significantly contribute to mitigating metabolic diseases related to obesity in the future.

## 2 Materials and methods

### 2.1 Preparation of *N. japonica* fruit extract

*Neoshirakia japonica* (Siebold & Zucc.) Esser [Euphorbiaceae] fruit extract was purchased from the Natural Product Central Bank (KPM025-071). The source fruits were originally collected from Ongnyong-myeon, Gwangyang-si, Jeollanam-do, Korea, in 2004 and then dried in the shade and concentrated, and a voucher specimen (KRIB 0003259) is kept in the herbarium of the Korea Research Institute of Bioscience and Biotechnology. 19 g of fruit powder was added to 1 L of 99.9% methyl alcohol (HPLC grade) and extracted using 30 ultrasonication cycles at room temperature in an ultrasonic extractor (SDN-900H, SD-Ultrasonic Co., Ltd., Korea), with 15 min of ultrasonication at 40 KHz and 1500 W followed by a 120 min standing period per cycle. Subsequently, the extract was filtered using a qualitative filter (No. 100, Hyundai Micro Co., Ltd., Seoul, Korea). The filtered extract underwent a first drying process in a water jacket-type dry oven (SCE&G, Kyung-gi, Korea) at 40 °C for 48 h. The dried sample was then subjected to a second drying step using a centrifugal vacuum concentrator (HANIL SCIENTIFIC INC. Seoul, Korea) at room temperature and 1,500 rpm under reduced pressure. To ensure complete drying, this step was extended up to 24 h as needed. For the third drying step, 300 μL of distilled water was added to each of the dried samples, followed by freezing in a deep freezer. The frozen samples were then lyophilized using a freeze-dryer (Ilsin BioBase, Korea) for up to 36 h, resulting in the final yield of 2.03 g of NJFE. The dried extract was immediately dissolved in dimethyl sulfoxide (DMSO; Sigma, Saint Louis, United States) at a concentration of 50 mg/mL and subsequently diluted to 2 mg/mL for experimental use. The 2 mg/mL solution was aliquoted into 30 µL portions and stored at −80 °C. Once thawed, it was neither re-frozen nor returned to −80 °C storage. For animal experiments, the dried extract was freshly dissolved in PBS immediately prior to use at a volume sufficient for 6 days of treatment (three injections, administered every other day). The prepared solution was stored at 4 °C and used as needed over a 6-day period. Any remaining volume after 6 days was discarded.

### 2.2 3T3-L1 cell cultures

3T3-L1 preadipocytes were purchased from ATCC (Manassas, United States). The cells were cultured in a growth medium consisting of 10% new bovine calf serum and 1% (v/v) penicillin-streptomycin (Gibco, Paisley, United Kingdom) in Dulbecco’s modified Eagle’s medium with high glucose (DMEM-H) (Gibco). When cells reached approximately 80% confluency, they were either passed using trypsin-EDTA (TE) (Gibco) or seeded into six-well plates.

### 2.3 Adipogenic differentiation of 3T3-L1 cells

When 3T3-L1 preadipocytes reached 100% confluency, they were maintained for an additional 2 days to arrest further growth. Then, the medium was replaced with 10% fetal bovine serum (FBS) and 1% (v/v) penicillin-streptomycin in DMEM-H supplemented with methylisobutylxanthine (0.5 mM), dexamethasone (0.25 mM), insulin (1 μg/mL), and indomethacin (0.125 nM) (MDI; Sigma), and the cells were cultured for 2 days. After this, the medium was replaced with 10% FBS, 1% (v/v) penicillin-streptomycin, and insulin (1 μg/mL) in DMEM-H to induce lipid accumulation. The cells were cultured in this medium for 6 days, with the medium changed every 2 days. Thus, the mature adipocyte differentiation induction process lasted for a total of 8 days.

### 2.4 RAW264.7 cell cultures

RAW264.7 macrophages were purchased from Korean Cell Line Bank (KCLB, Seoul, Korea). The cells were cultured in DMEM-H supplemented with 10% Fetal bovine serum (FBS) (Gibco) and 1% (v/v) penicillin-streptomycin. When the cells reached 70%–80% confluency in 100-mm culture dishes, they were seeded into six-well plates at a density of 1 × 10^6^ cells/well. After 24 h, the cells were treated with lipopolysaccharide (LPS; Sigma) at a concentration of 1 μg/mL to induce an inflammatory response.

### 2.5 Cell viability assessment

To evaluate the cytotoxicity of the NJFE, adipocytes were seeded in a 96-well plate at a density of 3 × 10^4^ cells/well and maintained for 24 h. The following day, when the cells reached 80% confluence, they were treated for 48 h with NJFE at concentrations ranging from 0.25 to 32 μg/mL or for the control group, the same volume of DMSO as used for the 32 μg/mL NJFE group. After treatment, 50 μL of cell counting kit-8 (CCK-8) solution (Dojindo Molecular Technologies, Kumamoto, Japan) was added to each well, and the plate was incubated at 37 °C for 1–2 h. The absorbance at 450 nm was measured using a microplate reader (Tecan, Männedorf, Switzerland).

### 2.6 Oil red O staining

At the end of differentiation, adipocytes were washed twice with PBS and fixed with 4% paraformaldehyde (Biosesang Inc., Yongin-si, Korea) for 1 h in the dark. After fixation, the cells were washed once with PBS. An oil red O (ORO) solution was prepared by diluting ORO (Sigma) with triple-distilled water at a 2:3 ratio, followed by centrifugation at 4,000 rpm for 20 min to collect the supernatant. The ORO solution was then added to each adipocyte sample, and the mixture was incubated for 30 min in the dark to allow staining. After staining, the cells were washed three times with triple-distilled water to remove any residual stain and then imaged under a microscope. After imaging, any remaining distilled water was removed by drying. To quantify the ORO content, 1 mL of isopropyl alcohol was added to each well to dissolve the stain, and the absorbance was measured at 450 nm using a microplate reader.

### 2.7 Real-time reverse-transcription polymerase chain reaction analysis

Total RNA was extracted from adipocytes and adipose tissue using RNAiso Plus (TRIzol; TaKaRa Bio, Kyoto, Japan), and the extracted RNA was converted into complementary DNA (cDNA) using a ReverTra Ace™ qPCR RT Master Mix kit (Toyobo, Kyoto, Japan). For the real-time reverse-transcription polymerase chain reaction (RT-qPCR), 1,000 ng of cDNA was mixed with SYBR Green master mix (Toyobo) and gene-specific primers, custom-designed and synthesized by Macrogen (Seoul, Korea) (Supplementary Table S1), and the reactions were performed using an iCycler iQ™ Real-Time PCR detection system (Bio-Rad Laboratories, Hercules, United States). The expression levels of the target genes were quantified relative to that of β-actin. The statistical analysis of the RT-qPCR results was performed using CFX Maestro software (Bio-Rad).

### 2.8 Western blot analysis

For adipocytes, proteins were extracted using RIPA buffer (Biosesang Inc.) supplemented with Xpert phosphatase inhibitor cocktail and Xpert protease inhibitor cocktail (Gendepot, Barker, TX, United States) solutions at 1:100 dilutions. For adipose tissue, protein extraction was performed using PRO-PREP™ (iNtRON Biotechnology, Seoul, Korea). Protein concentrations were adjusted to 30–60 μg using the Bradford assay (Bio-Rad), followed by the addition of 5X SDS-loading buffer (Biosesang Inc.) and boiling at 100 °C for 10 min. To separate proteins based on size, the boiled samples were loaded onto an SDS-polyacrylamide gel and subjected to electrophoresis. The separated proteins were then transferred onto a nitrocellulose membrane. To prevent the nonspecific binding of antibodies, the membrane was incubated in a blocking buffer (TBS-T with 5% skim milk) at room temperature for 1 h with shaking. Then, the membrane was incubated with the primary antibody overnight at 4 °C with shaking. The following day, the primary antibody was removed, and the membrane was washed three times in TBS-T for 5 min with shaking. The appropriate secondary antibody was then applied, and the membrane was incubated at room temperature for 1 h with shaking. The information on the antibodies used for the Western blot is provided in Supplementary Table S2. The secondary antibody was then removed, and the membrane was washed three times in TBS-T for 5 min with shaking. For band detection, the enhanced chemiluminescence (ECL) detection kit (GE Healthcare, Buckinghamshire, United Kingdom) was applied to the membrane, and images were captured using a FUSION Solo detector (Vilber Lourmat, Collégien, France). Band intensities were quantified with ImageJ software using the β-actin band’s intensity for normalization.

### 2.9 Enzyme-linked immunosorbent assay

The amount of TNFα secreted by cells was measured in the culture medium from stimulated RAW264.7 macrophages. The assay was performed according to the manufacturer’s protocol using the Mouse TNFα ELISA Kit–Quantikine (R&D Systems, Oxon, United Kingdom). Briefly, the cell supernatant was diluted 50-fold with Calibrator Diluent. The diluted samples were then added to an antibody-coated enzyme-linked immunosorbent assay (ELISA) plate and incubated in darkness at room temperature for 2 h with shaking. After incubation, the plate was washed with washing buffer, and the conjugate was added for further reaction. After another washing step, Substrate Solution was added, and the plate was incubated in darkness for 30 min with shaking. The reaction was then terminated by adding Stop Solution, and absorbance was measured at 450 nm using an ELISA reader (Tecan).

### 2.10 *In vivo* study

Male C57BL/6 mice were purchased at 6 weeks of age from HyoChang Science (Daegu, Korea). Before experimentation, the mice underwent a 1-week acclimation period. For the experiment, the mice were randomly divided into four groups: (1) a normal diet group (ND group), (2) a high-fat diet group (HFD group), (3) a group administered an HFD and 10 mg/kg/day of NJFE (NJFE10 group), and (4) a group administered an HFD and 20 mg/kg/day of NJFE (NJFE20 group). To induce obesity, the HFD (a rodent diet with 60 kcal% fat; Research Diets, NJ, United States) was provided for a total of 12 weeks. The composition of the HFD by weight was as follows: 34.89% fat, 26.23% protein, 25.56% carbohydrate, 6.46% fiber, and 6.46% minerals (Licholai et al., 2018). The ND and HFD group mice received PBS as a vehicle control, while the NJFE10 and NJFE20 group mice were orally administered NJFE diluted in PBS every other day. Body weight and food intake were measured every other day. At the end of the 12-week period, tissue and blood samples were collected for analysis. All animal experiments were conducted in accordance with the ethical guidelines of Kyungpook National University (Daegu, Korea) and were approved by the Institutional Animal Care and Use Committee (Approval No. KNU-2023-0208).

### 2.11 Hematoxylin and eosin staining

Epididymal and inguinal white adipose tissues (eWAT and iWAT) were fixed in 4% paraformaldehyde overnight. The fixed tissues were embedded in paraffin and sectioned at a thickness of 5 μm. Sections were then stained with hematoxylin and eosin (H&E). Stained adipose tissues were observed and imaged using a microscope (Leica Camera AG, Wetzlar, Hesse, Germany). The area (μm^2^) of lipid droplets in the images was quantified using the Adiposoft plug-in in ImageJ.

### 2.12 Immunohistochemical staining

Adipose tissue (eWAT) sections were deparaffinized, rehydrated through a graded ethanol series, and subjected to antigen retrieval in 10 mM citrate buffer (pH 6.0). The sections were then incubated overnight at 4 °C with anti-F4/80 antibody (macrophage marker; Cell Signaling Technology, MA, United States) diluted 1:200 in blocking solution. The following day, slides were washed with PBS and incubated with a secondary antibody for 1 h at room temperature. The staining signal was visualized using a DAB substrate, and images were captured using a light microscope (Leica Camera AG).

### 2.13 Intraperitoneal glucose tolerance test

Mice underwent an intraperitoneal glucose tolerance test (IP-GTT) during week 11 of the *in vivo* experiment to measure blood glucose levels. The day before the experiment, the mice fasted for at least 12 h. On the following day, all mice received an intraperitoneal injection of D-glucose at a dose of 1 g/kg at the same time, and blood glucose levels were measured 15, 30, 60, 90, 120 min post-injection using an Accu-Check EZ glucose monitor (Roche Molecular Biochemicals, IN, United States) via the tail vein.

### 2.14 Serum profiling

Blood was collected from mice via retro-orbital bleeding, and the samples were centrifuged at 3,500 rpm for 20 min at 4 °C to separate the plasma. The levels of glutamate oxaloacetate transaminase (GOT), glutamate pyruvate transaminase (GPT), blood urea nitrogen (BUN), creatinine (CREA), total cholesterol (T-CHO), high-density lipoprotein cholesterol (HDL-C), and low-density lipoprotein cholesterol (LDL-C) in the plasma were measured using an Olympus AU400 analyzer (Olympus Optical, Tokyo, Japan).

### 2.15 Fluorescence microscopy analysis of MitoTracker-stained mitochondria

To stain mitochondria, 3T3-L1 adipocytes were incubated with MitoTracker^®^ Orange (Thermo Fisher Scientific, Waltham, MA, United States). First, the dye was dissolved in DMSO to prepare a 1 mM stock solution. For staining, the stock solution was diluted in pre-warmed serum-free DMEM to a final working concentration of 25 nM. Cells in a six-well plate were washed twice with PBS, and 1 mL of the MitoTracker^®^ working solution was added to each well. The plates were then incubated for 30 min at 37 °C in a humidified CO_2_ incubator. After incubation, the cells were washed once with PBS and fixed with 4% paraformaldehyde (PFA) for 10 min at 37 °C. Following an additional PBS wash, stained cells were visualized using a fluorescence microscope (Leica Camera AG).

### 2.16 Flow cytometry

#### 2.16.1 MitoTracker-stained mitochondria

For mitochondrial staining, cells were harvested using trypsin-EDTA (Gibco) and pelleted by centrifugation at 3,000 rpm for 5 min. The cells were then washed twice with PBS and resuspended in a pre-warmed serum-free DMEM containing MitoTracker^®^ Orange (Thermo Fisher Scientific) at a concentration of 25 nM, and the mixture was incubated at 37 °C for 45 min in a humidified CO_2_ incubator. After incubation, the cells were centrifuged to remove any excess dye and resuspended in DMEM. The fluorescence intensity of the stained cells was measured using an Attune™ NxT Flow Cytometer (Thermo Fisher Scientific).

#### 2.16.2 Cell cycle analysis by propidium iodide (PI) staining

For cell cycle analysis, cells were treated with MDI and NJFE for 16 h. Subsequently, cells were dissociated into single-cell suspensions by treatment with Trypsin-EDTA (Gibco), followed by several washing steps. The single cells were then fixed with ice-cold 95% ethanol. After fixation, the cells were incubated with RNase A (100 μg/mL) at 37 °C in a CO_2_ incubator for 30 min, and subsequently resuspended in a staining solution containing propidium iodide (100 μg/mL). Flow cytometric analysis was performed using an Attune acoustic focusing cytometer (Thermo Fisher Scientific, Waltham, MA, United States). During acquisition, the voltages were set to 80 for forward scatter (FSC) and 260 for side scatter (SSC). After gating, a total of 1 × 10^4^ cells were analyzed using a 488 nm excitation laser and a 574/26 nm emission filter.

### 2.17 LC-MS analysis

NJFE detection was performed using an Alliance e2695 Separation Module (Waters Corporation, Milford, MA, United States) equipped with a Waters 2489 UV/Vis detector and a QDa mass detector (Waters Corporation, Milford, MA, United States). Chromatographic separation was carried out using an Acquity UPLC C18 column (250 mm × 3.0 mm, 5 μm particle size). Ammonium formate water was used as solvent A, and 0.1% formic acid in methanol was used as solvent B, with a flow rate of 0.6 mL/min. The gradient program consisted of an initial equilibration at 95% solvent A for 10 min, followed by a linear decrease of solvent A from 95% to 0% over 5–25 min, maintained at 0% solvent A from 25 to 30 min, then increased back to 95% solvent A between 30 and 30.01 min, and finally re-equilibrated at 95% solvent A from 30.01 to 35 min. The NJFE sample was injected at a concentration of 10 ppm. Mass spectrometry was performed in negative electrospray ionization mode, scanning from 50 to 800 m/z. The probe temperature was maintained at 400 °C, and the source temperature was set to 120 °C. Collision energy was gradually increased from 10 to 40 V, and the capillary voltage in positive mode was set to 0.8 kV. The LC-MS analysis results and detailed conditions are shown in Figure 7.

### 2.18 Statistical analysis

All experiments were performed with three technical and three biological replicates. Data are presented as the mean ± the standard deviation for *in vitro* experiments and as the mean ± the standard error of the mean for *in vivo* experiments. Statistical significance was determined using one-way ANOVAs in IBM SPSS Statistics 27 and GraphPad Prism 9 (GraphPad Software, San Diego, CA, United States). A *p*-value of less than 0.05 was considered statistically significant.

## 3 Results

### 3.1 NJFE produces anti-adipogenic effects in HFD-fed obese mice without *in vivo* toxicity

**FIGURE 1:**
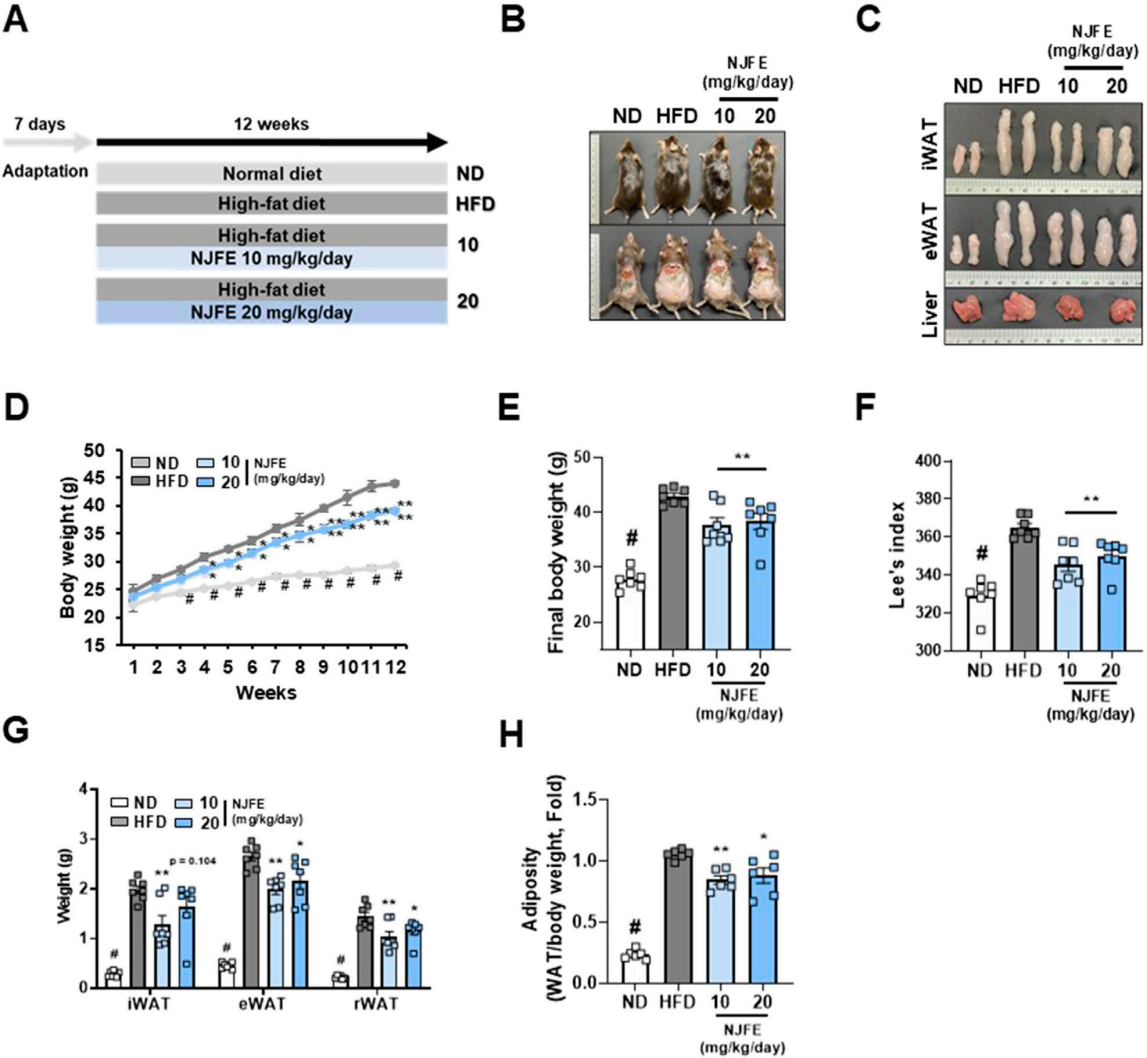
Effects of NJFE on lipid accumulation in high-fat diet (HFD)-induced obese mice. **(A)** The experimental plan for the *in vivo* studies. The treatment duration was 12 weeks. **(B)** Images of the mouse’s overall appearance and the intra-abdominal area. **(C)** Representative images of the inguinal (iWAT) and epididymal (eWAT) white adipose tissues and livers of mice. **(D)** The weekly body weight gains of the mice over the 12-week experimental period. **(E)** The final body weights of the mice. Mice fasted for at least 12 h prior to measurement, and their body weight was recorded immediately before sacrifice. **(F)** Lee’s index of the mice, calculated based on body weight and length. **(G)** The weight of mouse adipose tissues, measured from three areas: iWAT (subcutaneous fat), eWAT, and rWAT (visceral WAT). **(H)** The adiposity of the mice calculated based on mouse body weight and adipose tissue weight. All data are presented as means ± standard deviations. Statistical significance is denoted using symbols: “#” indicates a statistically significant difference (*p* < 0.05) between the control groups, ND and HFD, and “*” and “**” indicate *p*-values of <0.05 and <0.01 for comparisons between the NJFE-treated and HFD mice.

We used a mouse model of obesity induced by a 12-week HFD to evaluate the anti-obesity effects of NJFE. Mice were orally administered by NJFE along with HFD. Mice were randomly assigned to groups administered an ND (Normal diet), an HFD (High-fat diet), an HFD plus 10 mg/kg/day of NJFE or an HFD plus 20 mg/kg/day of NJFE (Figure 1A). To confirm that the doses used in this study did not induce *in vivo* toxicity, we evaluated changes in the weights of the liver, spleen, and kidneys, as well as systemic toxicity markers. NJFE administration at all tested doses did not cause significant alterations in organ weights. In addition, serum biochemical analyses showed no significant changes in the hepatic toxicity markers GOT (aspartate aminotransferase) and GPT (alanine aminotransferase) or the renal toxicity markers BUN (blood urea nitrogen) and CREA (creatinine) compared with the control group. These results indicate that the doses of NJFE used in this study did not cause systemic toxicity in mice (Supplementary Figures S1A,B). However, the NJFE treatments significantly inhibited the accumulation of both subcutaneous and visceral fat induced by the HFD, as observed visually. Furthermore, the HFD-induced expansion of adipose tissue, including iWAT and eWAT, was also suppressed, and it was confirmed that hepatic fat accumulation induced by the HFD was significantly reduced by the NJFE treatments (Figures 1B,C). Compared to the HFD group, the NJFE treatments produced a significant weight reduction effect starting in the fourth week, and the final body weight at 12 weeks was significantly lower (Figures 1D,E). To accurately assess obesity in rodents, we calculated Lee’s Index, which is based on body weight and length, and Lee’s index was significantly lower in the NJFE treatment groups than in the HFD group (Figure 1F). In addition to weight reduction, NJFE treatments significantly decreased the weights of iWAT, eWAT, and rWAT (retroperitoneal WAT) (Figure 1G). This led to a reduction in adiposity, as reflected by decreases in the adipose tissue weights of mice relative to their body weights (Figure 1H). The weight loss effect of NJFE in obese mice was not due to changes in food intake (Supplementary Figure S1C). Therefore, the results of this study suggest that NJFE inhibits weight gain and adipose tissue expansion induced by HFD.

### 3.2 NJFE inhibits adipocyte hypertrophy, dyslipidemia, and glucose intolerance in HFD-fed obese mice

**FIGURE 2:**
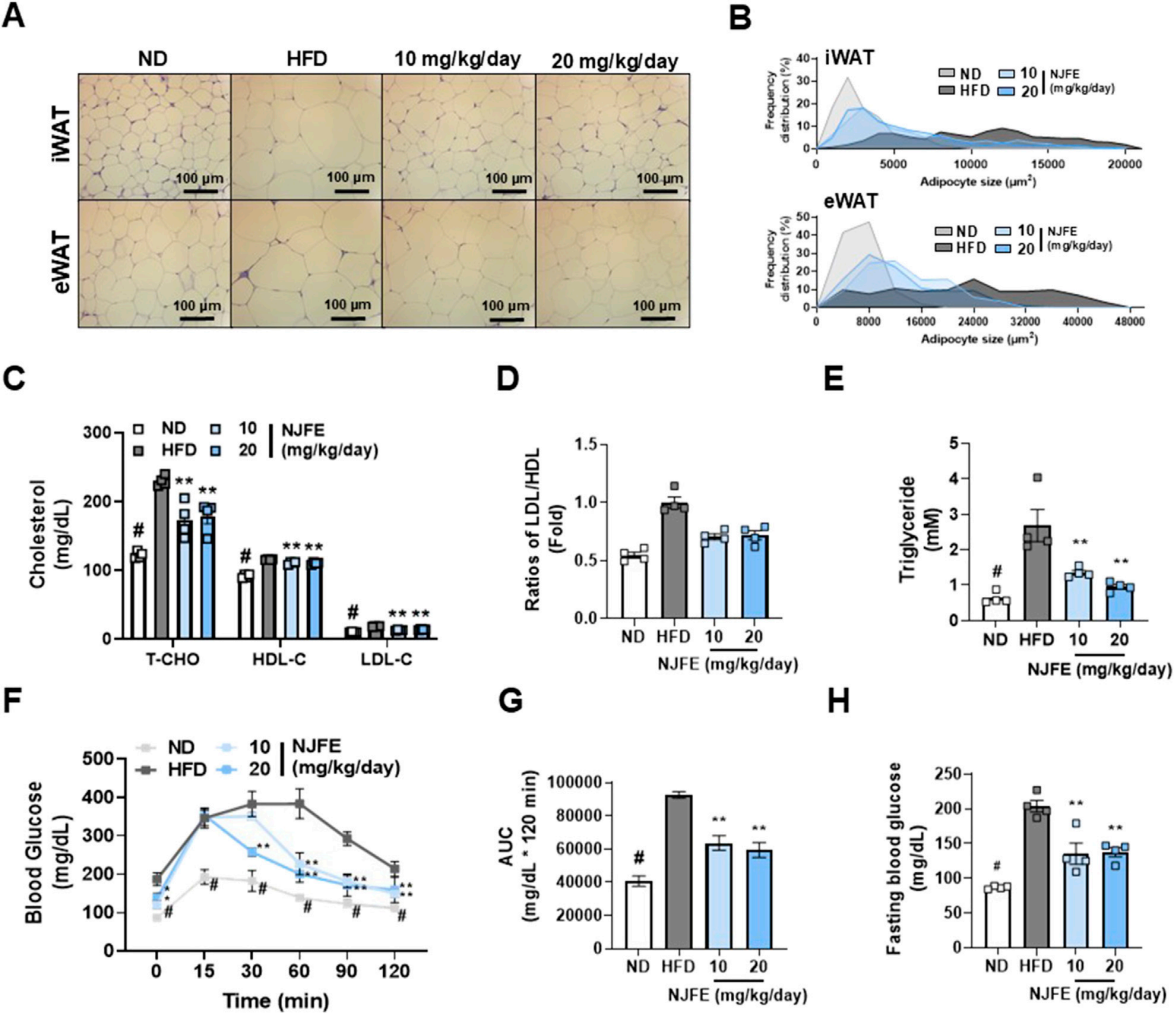
Effects of NJFE on adipocyte hypertrophy, dyslipidemia, and glucose intolerance in high-fat diet (HFD)-induced obese mice. **(A)** H&E staining images showing the morphology of lipid droplets in epididymal (eWAT) and inguinal (iWAT) white adipose tissues. Images were taken at 400× magnification. The scale bar represents 100 μm. **(B)** A graph showing the frequency distribution of lipid droplet areas (µm^2^) in iWAT (Top) and eWAT (Bottom), expressed as a percentage of the total quantified lipid droplet frequency distribution. **(C)** The serum cholesterol levels of the mice: T-CHO (total cholesterol), HDL-C (high-density lipoprotein cholesterol), and LDL-C (low-density lipoprotein cholesterol). **(D)** The LDL-C/LDL-C ratios of the mice. The LDL-C level was divided by the HDL-C level and expressed as a fold change relative to the mean LDL-C/LDL-C value of the control mice. **(E)** Serum triglyceride levels. **(F)** A GTT graph showing changes in the blood glucose levels of the mice. Glucose was injected at 0 min, and blood glucose levels were then measured at 30-min intervals through the tail vein. **(G)** An AUC (area under the curve) graph representing the quantified areas under the GTT curves in **(F)**. **(H)** Fasting blood glucose levels of the mice. Blood was collected through the tail vein of mice after 12 h of fasting. All data are presented as means ± standard deviations. Statistical significance is denoted using symbols: “#” indicates a statistically significant difference (*p* < 0.05) between the control groups, ND and HFD, and “*” and “**” indicate *p*-values of <0.05 and <0.01 for comparisons between the NJFE-treated and HFD mice.

We investigated whether NJFE has effects beyond weight loss and reductions in adipose tissue mass. Specifically, whether it has the potential to improve obesity-induced adipocyte hypertrophy, dyslipidemia, and insulin resistance. Compared to the HFD group, the NJFE treatment groups showed significant reductions in lipid droplet area in both iWAT and eWAT (Figures 2A,B). Additionally, we observed that the serum levels of T-CHO, LDL-C, and HDL-C were lower in the NJFE treatment groups than in the HFD group (Figure 2C). To determine whether the decrease in HDL-C is due to the reduction in T-CHO, we analyzed the LDL/HDL ratio. The LDL/HDL ratio is commonly used as an indicator of cardiovascular disease risk and effectively quantifies the balance between good cholesterol and bad cholesterol in the blood. We observed a significant decrease in the LDL/HDL ratio in the NJFE-treated groups compared to the HFD group (Figure 2D). Similarly, the NJFE-treated groups also showed a significant decrease in serum triglyceride levels (Figure 2E). Regarding insulin resistance and glucose intolerance, when glucose was administered, mice in the NJFE-treated groups began to recover from the glucose-induced blood sugar increase starting 30–60 min after administration, whereas HFD-group mice did not start to recover for up to 60 min after administration. The difference in blood sugar reduction between the NJFE 20 0 group and the HFD group became significant starting at 30 min. A significant difference in blood glucose levels between the NJFE 10 group and the HFD group began at 60 min (Figures 2F,G). We also observed that the basal blood glucose levels after fasting were significantly lower in the NJFE-treated groups than in the HFD group (Figure 2H). In summary, these results suggest that NJFE significantly improves the adipocyte hypertrophy, dyslipidemia, insulin resistance, and glucose intolerance caused by diet-induced obesity.

### 3.3 NJFE alleviates adipose tissue inflammation caused by HFD-induced obesity

**FIGURE 3:**
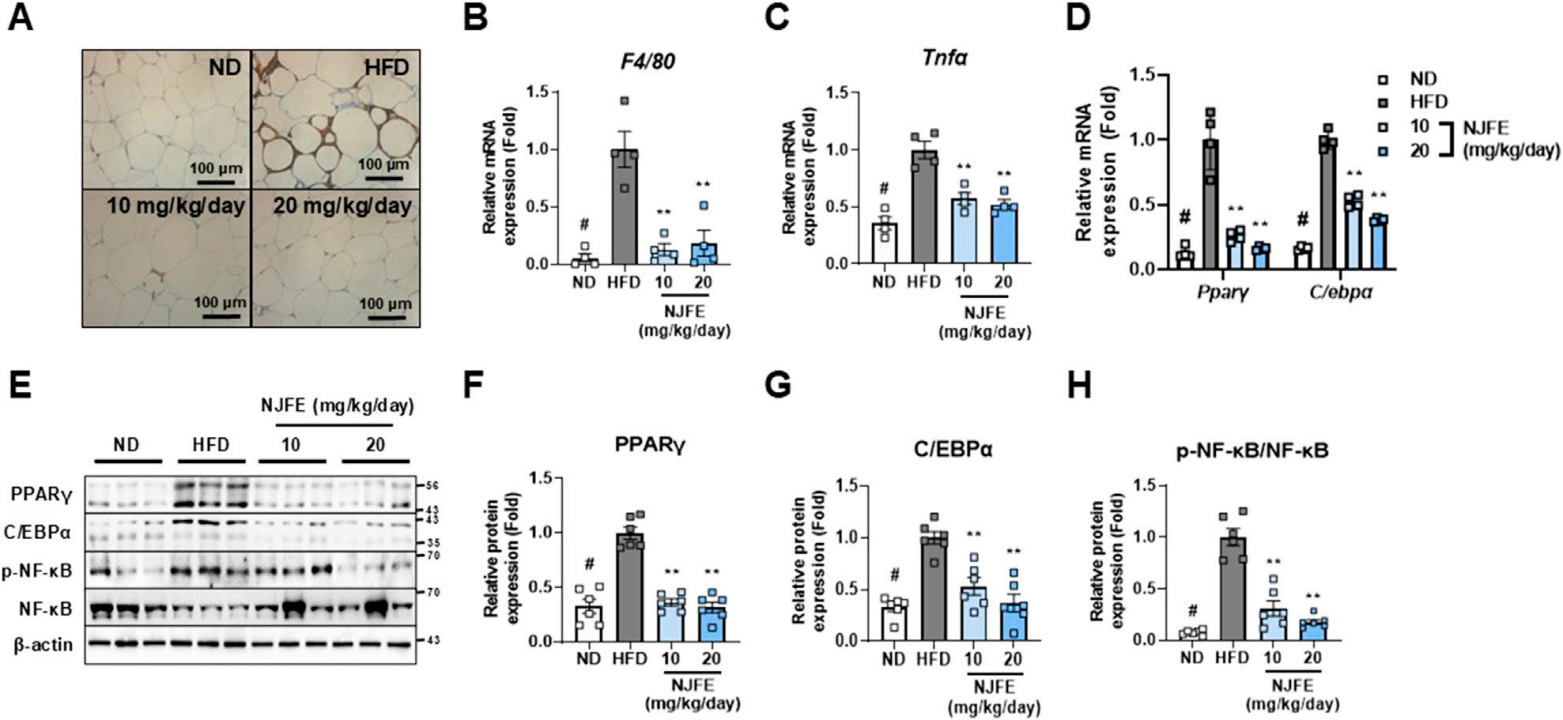
Effects of NJFE on adipose tissue inflammation in high-fat diet (HFD)-induced obese mice. **(A)** F4/80 immunohistochemistry images of epididymal white adipose tissue (eWAT). Images were taken at 400× magnification. The scale bar represents 100 μm. The mRNA levels of two genes in eWAT, measured using RT-qPCR: **(B)** *F4/80* and **(C)** *Tnfα*. **(D)** Expression levels of two adipogenesis-related genes in eWAT at the mRNA level. **(E)** Protein expression levels of adipogenesis-related proteins, PPARγ and C/EBPα, and phosphorylated NF-κB in eWAT, measured via Western blot analysis. The numbers on the right indicate the molecular weights of the protein bands (kDa). **(F)** A graph of PPARγ protein expression quantified based on Western blot band intensities. **(G)** Graph of C/EBPα protein expression quantified based on Western blot band intensities. **(H)** Graph of NF-κB phosphorylation levels quantified based on Western blot band intensities. The phosphorylation level was calculated by dividing the p-NF-κB band intensity by the NF-κB band intensity. All Western blot band intensities and RT-qPCR results were normalized using β-actin and are presented as fold changes relative to the expression levels in the control (HFD) group. All data are presented as means ± standard deviations. Statistical significance is denoted using symbols: “#” indicates a statistically significant difference (*p* < 0.05) between the control groups, ND and HFD, and “**” indicates a *p*-value <0.01 for comparisons between the NJFE-treated and HFD mice.

Building on our previous finding that NJFE inhibits HFD-induced obesity and mitigates adipocyte hypertrophy, dyslipidemia, and glucose intolerance, we examined whether NJFE also alleviates obesity-induced adipose tissue inflammation. The protein F4/80 is specifically expressed in tissue-resident macrophages and is used as a marker to assess the presence and activation status of macrophages in adipose tissue. Obesity is characterized by a chronic inflammatory state in adipose tissue in which recruited adipose tissue macrophages induce inflammation and act as mediators of insulin resistance (Lumeng et al., 2007). Therefore, we first investigated whether NJFE inhibits macrophage recruitment in the inflammatory environment induced by obesity by examining F4/80 expression in eWAT. As confirmed by immunohistochemistry, the HFD induced elevated F4/80 protein expression, which was reduced by the NJFE treatments, and this reduction was consistent with decreases in *F4/80* gene expression (Figures 3A,B). This led to a decrease in *Tnfα* expression in the NJFE treatment groups compared to the HFD group (Figure 3C). Additionally, we analyzed the NJFE treatments’ effects on adipogenesis markers and the phosphorylation levels of NF-κB in eWAT. The transcription factors PPARγ and C/EBPα are well-known master regulators involved in adipocyte differentiation and lipid synthesis, and decreases in their expression suggest a reduction in adipogenesis (Rosen, 2005). The NF-κB pathway is a prominent mechanism that induces inflammation and is a strong mediator of insulin resistance (Shoelson et al., 2003). We observed increased mRNA and protein expression levels of PPARγ and C/EBPα in HFD-fed obese mice, and this increase was significantly reduced in NJFE treatment-group mice (Figures 3D–G). At the same time, HFD-induced phosphorylation of NF-κB was also significantly decreased by the NJFE treatments (Figures 3E,H). Based on these findings, we propose that NJFE not only alleviates obesity through the inhibition of adipogenesis-related gene expression but also suppresses obesity-induced macrophage recruitment and inflammation in adipose tissue.

### 3.4 NJFE inhibits adipogenesis by regulating adipogenic differentiation-related gene expression in 3T3-L1 adipocytes

**FIGURE 4:**
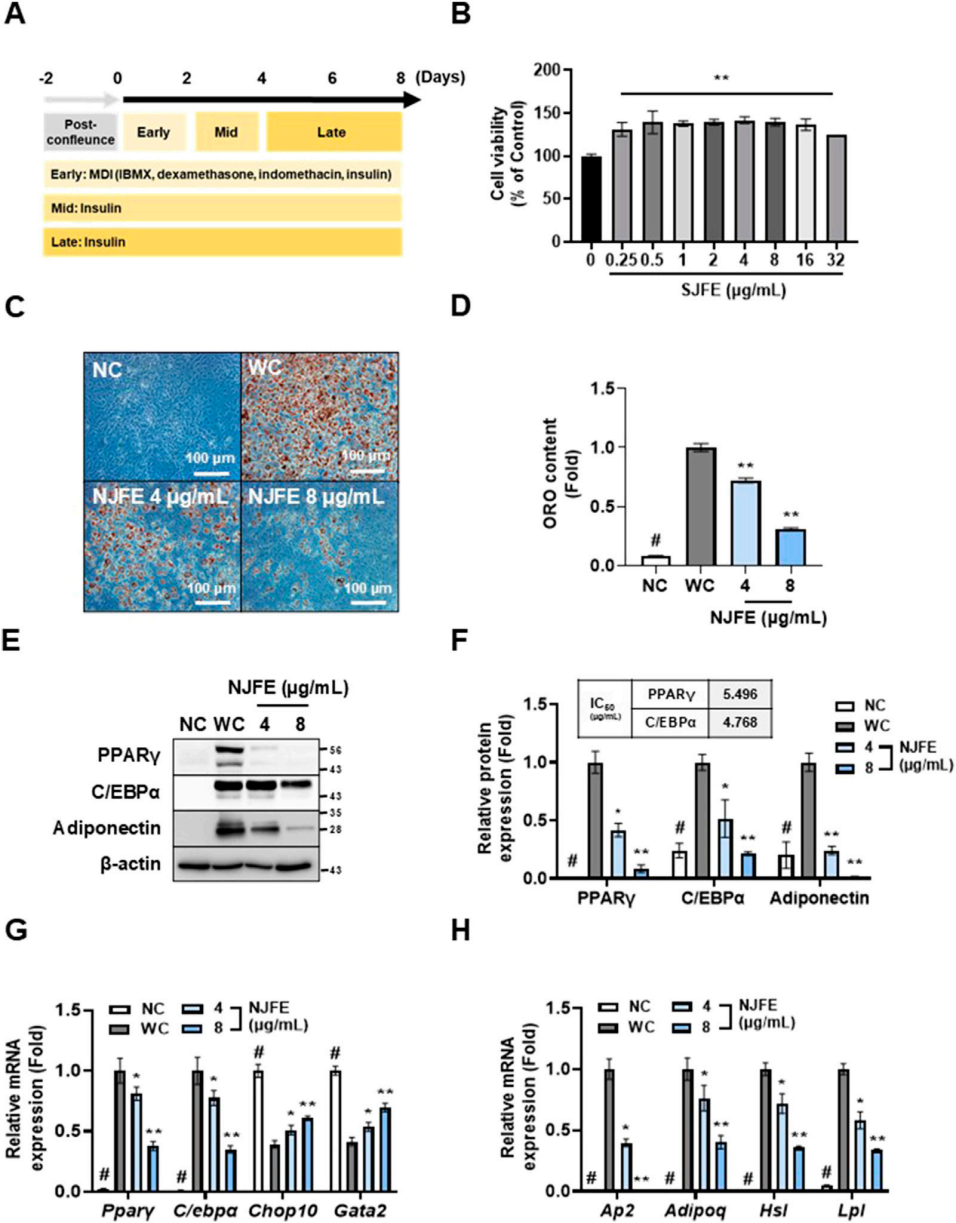
The inhibitory effect of NJFE on adipogenesis is mediated through the regulation of adipogenic differentiation-related gene expressions in 3T3-L1 adipocytes. *In vitro* experimental groups included NC (non-differentiated cells), WC (white adipocytes), and white adipocytes treated with NJFE at 4 or 8 μg/mL. **(A)** A schematic representation of the adipocyte differentiation protocol. Differentiation was induced by treating preadipocytes with MDI in the early stage, followed by insulin treatment during the mid and late stages. **(B)** The cell viability of 3T3-L1 cells after NJFE treatment, as assessed using the CCK-8 assay. For the control (0), the same volume of DMSO as that used in the 32 μg/mL NJFE group was applied. **(C)** Oil red O staining of differentiated adipocytes. Representative images of NC, WC, and NJFE-treated (4 and 8 μg/mL) cells are shown. Images were taken at 200× magnification. The scale bar represents 100 μm. Lipid accumulation is indicated by red staining. **(D)** A graph of oil red O (ORO) staining intensity, measured as absorbance at 450 nm. The bars represent relative lipid accumulation. **(E)** A Western blot membrane showing the protein expression levels of PPARγ, C/EBPα, and adiponectin. The numbers on the right indicate the molecular weights of the protein bands (kDa). **(F)** A graph of PPARγ, C/EBPα, and adiponectin protein expression levels quantified based on Western blot band intensities. The IC_50_ values (µg/mL) for PPARγ and C/EBPα are indicated at the top of the graph. Relative mRNA levels, based on RT-qPCR analysis, of **(G)** transcription factors related to adipocyte differentiation and **(H)** markers associated with adipocyte maturation and lipid accumulation. All Western blot band intensities and RT-qPCR results were normalized using β-actin and are presented as fold changes relative to the expression levels in the control (WC) group. All data are presented as means ± standard deviations. Statistical significance is denoted using symbols: “#” indicates a statistically significant difference (*p* < 0.05) between the control groups, NC and WC, and “*” and “**” indicate *p*-values of <0.05 and <0.01 for comparisons between the NJFE-treated adipocytes and WC.

To assess the reproducibility of NJFE’s *in vivo* anti-obesity and obesity complication-ameliorating effects, we conducted *in vitro* experiments using 3T3-L1 adipocytes. Adipocyte differentiation was conducted over a total of 8 days, following the induction protocol shown in Figure 4A. NJFE showed no cytotoxicity up to 32 μg/mL. Therefore, we used the lowest effective concentrations, 4 and 8 μg/mL, in adipocyte experiments (Figure 4B). Treating cells with NJFE throughout the entire differentiation period led to a dose-dependent decrease in adipocyte differentiation and lipid accumulation (Figures 4C,D). To investigate whether this reduction was due to decreases in the expression of adipogenesis-related genes, we examined the protein levels of two key transcription factors regulating adipogenesis, PPARγ and C/EBPα, and the lipid accumulation marker adiponectin. The expression of these three proteins decreased in a dose-dependent manner with NJFE treatment. The IC_50_ values for PPARγ and C/EBPα were 5.496 and 4.768 μg/mL, respectively (Figures 4E,F). Additionally, we analyzed the gene expression levels of four transcription factors, two that promote adipocyte differentiation, *Pparγ* and *C/ebpα*, and two that inhibit it, *Chop10* and *Gata2*. *Chop10* suppresses adipocyte differentiation by inactivating C/EBPβ (Huang et al., 2008), while *Gata2* acts as a negative regulator of adipogenesis by interacting with and repressing PPARγ (Tong et al., 2000). The NJFE treatments reduced *Pparγ* and *C/ebpα* expression in a dose-dependent manner while increasing *Chop10* and *Gata2* expression. Beyond its regulation of transcription factors related to adipocyte differentiation, NJFE also significantly reduced the expression of the adipocyte maturation and lipid accumulation marker genes *Ap2*, *Adipoq*, *Hsl*, and *Lpl* (Figures 4G,H). In addition, we examined IRβ and IRS1 as upstream targets of NJFE. These molecules are key metabolites of the insulin signaling pathway and play a crucial role in adipocyte differentiation by mediating the insulin-induced expression of major adipogenic transcription factors such as PPARγ and C/EBPα (Miki et al., 2001). 8 μg/mL of NJFE treatment significantly reduced the expression of both IRβ and IRS1 (Supplementary Figure S4A). Therefore, the downregulation of IR and IRS1 may serve as a molecular mechanism by which NJFE inhibits adipocyte differentiation. Collectively, these findings suggest that NJFE inhibits adipocyte differentiation and lipid accumulation by modulating key transcription factors involved in adipogenesis.

### 3.5 NJFE effectively inhibits the initiation of adipocyte differentiation

After confirming that NJFE inhibits adipocyte differentiation, we investigated at which stage of adipocyte differentiation NJFE exerts its strongest inhibitory effect. Adipocyte differentiation was divided into three stages: early (0–2 days), middle (2–4 days), and late (4–6 days). Accordingly, NJFE was administered at various points corresponding to all combinations of these stages (Figure 4A; Supplementary Figure S2A). Lipid accumulation was significantly reduced when the NJFE was applied during the early differentiation stages. However, administration in the middle or late stages resulted in lower reductions in lipid accumulation if NJFE was not applied in the early stage, regardless of the treatment duration (Supplementary Figures S2B,C). Furthermore, under the same experimental design, we investigated the mRNA expression of *Pparγ*. The expression of *Pparγ* was significantly more reduced in the groups treated with NJFE during the early stage (B,C,D,H) than in those not treated during the early stage (E,F,G). Therefore, this result supports the ORO staining data (Supplementary Figure S2D).

The early stage of adipocyte differentiation is the most critical, as it involves the regulation of the cell cycle through the mitotic clonal expansion of preadipocytes, initiating irreversible differentiation (Audano et al., 2022). Before differentiation induction, most preadipocytes that reach confluence remain in the G1 phase and cease growth. However, when treated with the differentiation inducer MDI, the cells re-enter the cell cycle and undergo one or two rapid divisions, a process known as mitotic clonal expansion (MCE). Previous studies have shown that MCE begins approximately 16 h after MDI treatment (Lee et al., 2023). In the early stage of adipocyte differentiation, C/EBPβ and C/EBPδ function as key transcription factors (Clarkson et al., 1995). To evaluate the effect of NJFE at this stage, 3T3-L1 preadipocytes were induced to differentiate for 16 h while simultaneously treated with NJFE. Subsequent analysis revealed that NJFE treatment significantly reduced the expression levels of *C/ebpβ* and *C/ebpδ* compared to the WC (Supplementary Figure S2E). In terms of the cell cycle, pre-differentiated cells (NC) were largely distributed in the G1 phase, and MDI treatment decreased the G1 phase distribution and increased the S and G2 phase distributions. However, when NJFE was treated, the G1 phase distribution increased and the S and G2 phase distributions decreased compared to WC, and this was dose-dependent. Therefore, NJFE delays the onset of MCE, which occurs when adipocyte differentiation is initiated (Supplementary Figures S2F,G). In conclusion, these findings highlight the anti-obesity effect of NJFE, which stems from its robust inhibition of the initial gate-keeping steps of adipocyte differentiation, thereby preventing fat cell hypertrophy.

### 3.6 NJFE suppresses macrophage-induced inflammatory responses in RAW264.7 cells

**FIGURE 5:**
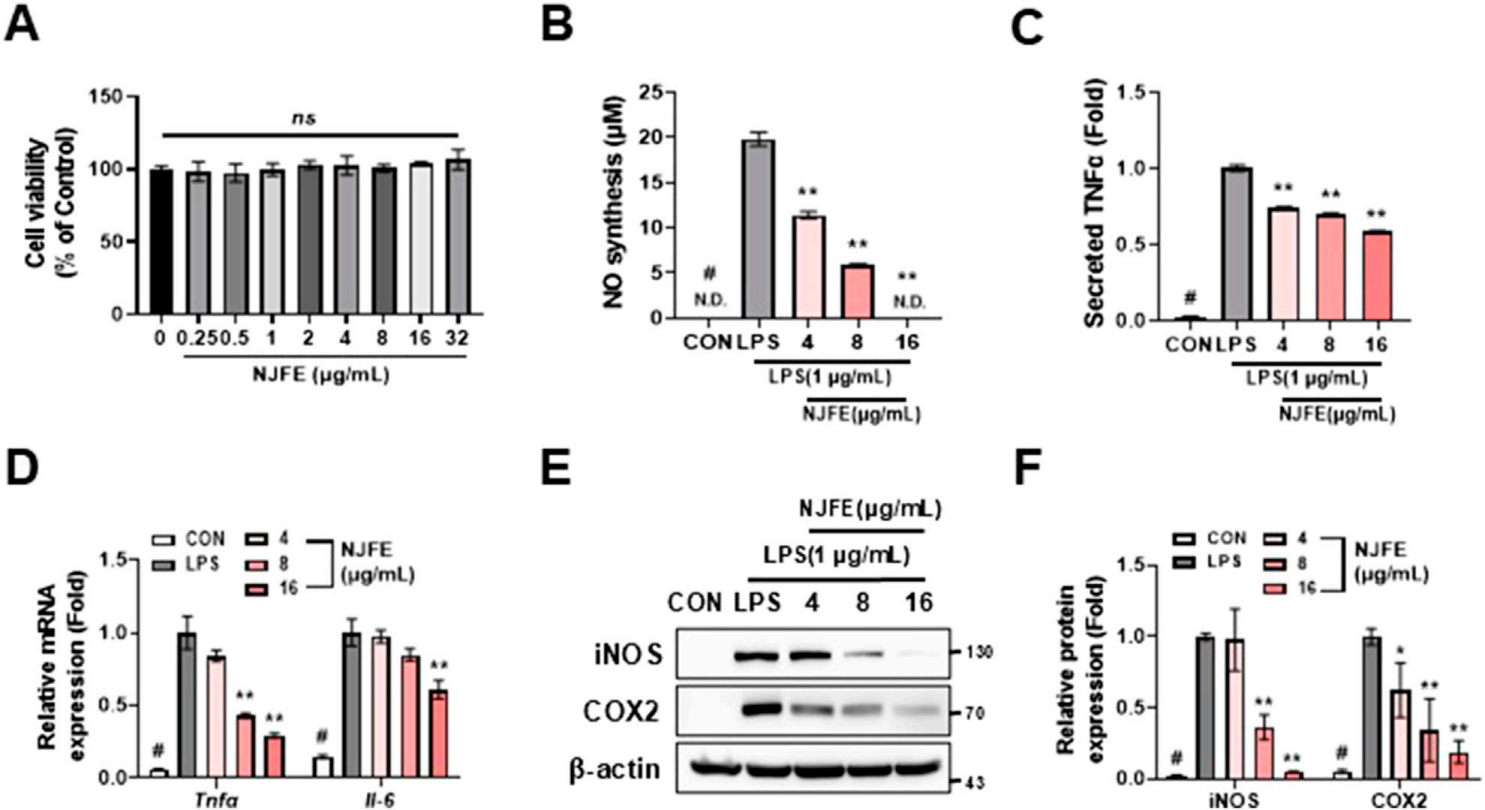
The anti-inflammatory effect of NJFE on macrophage-mediated inflammatory responses. **(A)** Cell viability of RAW264.7 cells after NJFE treatment, as assessed using the CCK-8 assay. For the control (0), the same volume of DMSO as that used in the 32 μg/mL NJFE group was applied. **(B)** The concentrations (μM) of nitric oxide (NO) secreted by RAW264.7 macrophages in the cell culture medium. **(C)** The concentrations of TNFα secreted by RAW264.7 macrophages in the cell culture medium. The values are presented as fold change relative to the control (LPS). **(D)** The relative mRNA levels of *Tnfα* and *Il-6* in RAW264.7 macrophages, measured using RT-qPCR. **(E)** A Western blot membrane showing the protein expression levels of iNOS and COX2. The numbers on the right indicate the molecular weights of the protein bands (kDa). **(F)** A graph of iNOS and COX2 protein expression levels quantified based on Western blot band intensities. Western blot band intensities and RT-qPCR results were normalized using β-actin and are presented as fold changes relative to the expression levels in the control (LPS) group. All data are presented as means ± standard deviations. Statistical significance is denoted using symbols: “#” indicates a statistically significant difference (*p* < 0.05) between the control groups, CON and LPS, and “*” and “**” indicate *p*-values of <0.05 and <0.01 for comparisons between the NJFE-treated and LPS cells.

Adipose tissue, being a major site of inflammation in obese individuals, should be considered not only as a storage reservoir for lipids but also as an immune organ. Inflammation within adipose tissue is driven by adipose tissue-associated macrophages, and the shift towards an inflammatory environment leads to insulin resistance (O'Rourke, 2009). Therefore, we aimed to confirm that NJFE alleviates macrophage-derived inflammation. The NJFE did not exhibit cytotoxicity up to a concentration of 32 μg/mL in RAW264.7 macrophages (Figure 5A). In subsequent tests, NJFE treatments 4–16 μg/mL reduced the levels of nitric oxide and TNFα released by lipopolysaccharide (LPS)-activated macrophages (Figures 5B,C). Additionally, the expression of *Tnfα* and *Il-6*, which increased upon LPS treatment, was decreased by the NJFE treatments in a dose-dependent manner. We also evaluated the effect of NJFE on the expression of the proteins iNOS and COX2, which were upregulated in the macrophages upon LPS-induced inflammation. The NJFE treatments reduced both iNOS and COX2 expressions in a dose-dependent manner. Taken together, these results are evidence that NJFE alleviates LPS-induced macrophage inflammation and the release of inflammatory cytokines by macrophages *in vitro*.

### 3.7 NJFE suppresses adipocyte inflammation induced by lipogenesis and an activated macrophage-conditioned medium

**FIGURE 6:**
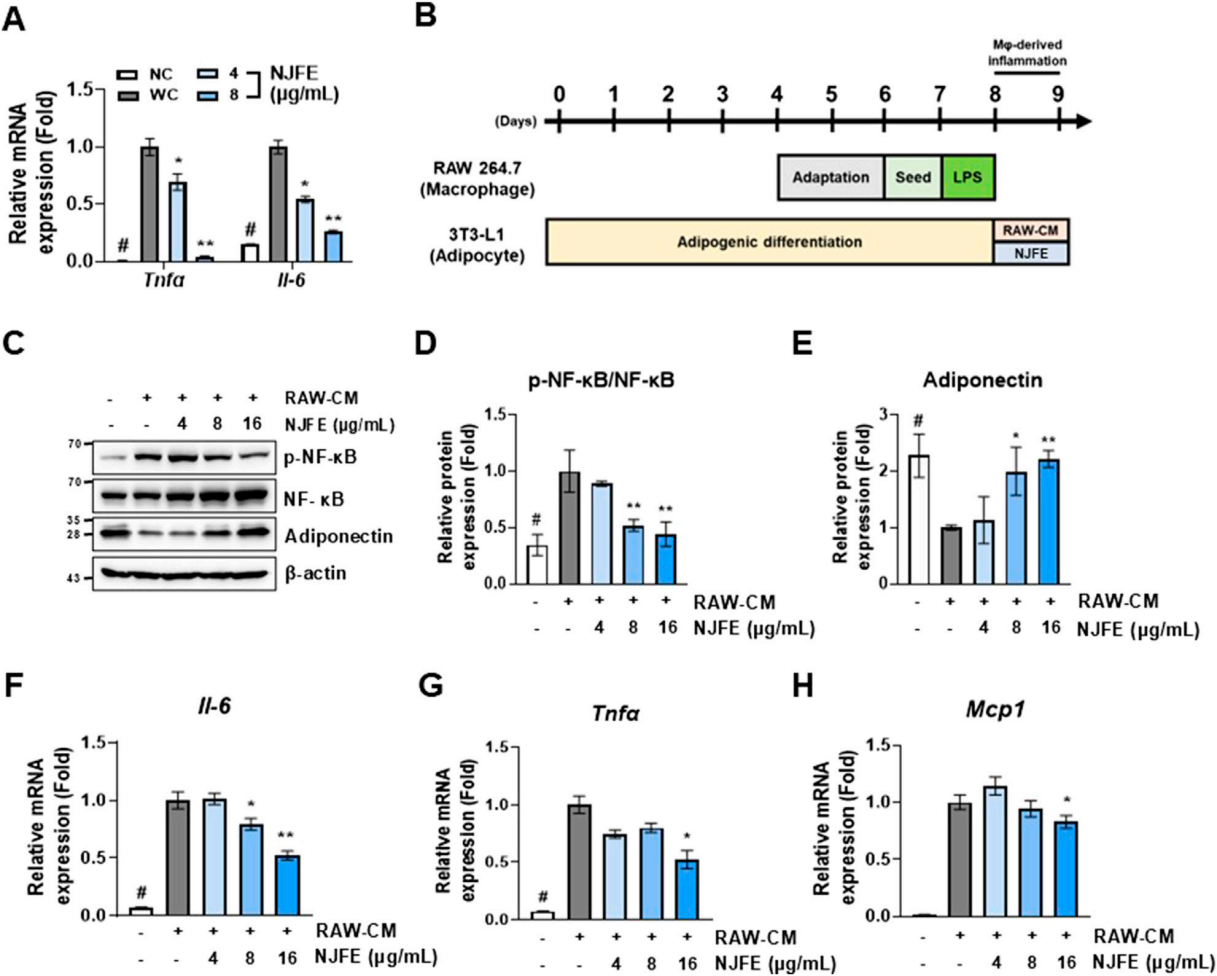
The alleviative effects of NJFE on adipocyte inflammation induced by lipogenesis and an activated macrophage-conditioned medium. **(A)** The relative mRNA levels of *Tnfα* and *Il-6* in 3T3-L1 adipocytes. **(B)** A schematic detailing the treatment of 3T3-L1 cells with a RAW264.7 macrophage-conditioned medium (RAW-CM) in cell culture experiments. **(C)** A Western blot membrane showing the phosphorylation level of NF-κB and adiponectin protein expression. The numbers on the left indicate the molecular weights of the protein bands (kDa). **(D** and **E)** Graphs of **(D)** NF-κB phosphorylation levels and **(E)** adiponectin expression levels, both quantified based on Western blot band intensities. The relative mRNA levels, based on RT-qPCR analysis, of **(F)** *Il-6*, **(G)** *Tnfα*, and **(H)** *Mcp1* in RAW-CM-treated 3T3-L1 adipocytes. Western blot band intensities and RT-qPCR results were normalized using β-actin and are presented as fold changes relative to line 2. All data are presented as means ± standard deviations. Statistical significance is denoted using symbols: “#” indicates a statistically significant difference (*p* < 0.05) between the control groups, lines 1 and 2, and “*” and “**” indicate *p*-values of <0.05 and <0.01 for compared to line 2.

Based on our findings that the NJFE treatments inhibited macrophage activation and macrophage-induced inflammatory responses (Figure 5) and alleviated lipogenesis-induced inflammation in adipocytes (Figure 6A), we examined whether NJFE could suppress inflammation induced by activated macrophages in adipocytes within an obese environment. In an obese environment, serum levels of free fatty acids, cholesterol, and LPSs increase, leading to a rise in macrophage numbers, which can comprise up to 40% of the cells in adipose tissue and promote polarization to the M1 phenotype, triggering an inflammatory response (Castoldi et al., 2016; Weisberg et al., 2003). This occurs because elevated serum LPS levels, like those seen in obesity, are associated with changes in the gut microbiota. As LPSs are metabolites of the cell walls of Gram-negative bacteria, they act as a key factor in activating macrophages and promoting inflammation (Caricilli et al., 2011).

Based on this, we induced RAW264.7 macrophage activation with LPS, isolated the resulting culture medium (RAW-CM), and then used it to treat 3T3-L1 adipocytes to mimic the obesity-induced inflammatory environment in adipose tissue (Figure 6B). The RAW-CM treatment increased NF-κB activation in adipocytes, but this activation was attenuated when treated with NJFE. Additionally, NJFE treatment mitigated RAW-CM-induced reductions in adiponectin expression in adipocytes (Figures 6C–E). Adiponectin, a hormone derived from adipose tissue, plays a protective role by suppressing the development of obesity-related diseases, and its expression is reduced in obesity (Ouchi and Walsh, 2007). Moreover, NJFE alleviated RAW-CM-induced increases in the expression of genes encoding inflammatory cytokines, such as *Il-6*, *Tnfα*, and *Mcp1*, in adipocytes. Notably, *Mcp1*, primarily produced by macrophages, is closely associated with macrophage infiltration into adipose tissue in the obese environment (Kanda et al., 2006). In summary, NJFE not only alleviates lipogenesis-induced inflammation in adipocytes but also effectively suppresses the inflammatory response induced by obesity-activated macrophages in adipocytes.

### 3.8 NJFE improves mitochondrial function in 3T3-L1 adipocytes

Obesity is characterized by increased oxidative stress and exacerbated inflammation, accompanied by immune cell infiltration into adipocytes. These changes lead to the impairment of the mitochondrial electron transport chain and a reduction in mitochondrial biogenesis. This obesity-induced mitochondrial dysfunction, in turn, results in metabolic alterations in fatty acid oxidation and lipolysis as well as insulin resistance (de Mello et al., 2018). Therefore, if NJFE can improve mitochondrial function, it may provide additional benefits in alleviating obesity and its complications. Compared to preadipocytes (NC), adipocytes subjected to lipogenesis for a total of 8 days (WC) exhibited reduced mitochondrial biogenesis due to the inflammation intrinsically caused by lipid synthesis and accumulation. However, when NJFE was administered during differentiation, mitochondrial biogenesis was improved (Supplementary Figures S3A,B). Additionally, given that CPT1 (Carnitine Palmitoyltransferase 1) is a key regulator of fatty acid oxidation in mitochondria, we examined its expression. As with mitochondrial biogenesis, CPT1 expression was reduced by lipogenesis, but NJFE treatment during differentiation restored it (Supplementary Figure S3C). Collectively, our findings suggest that NJFE not only alleviates obesity and obesity-induced inflammation in adipose tissue but also enhances mitochondrial function in adipocytes, potentially offering additional therapeutic benefits for obesity and obesity-related complications.

### 3.9 Characterization of the chemical fingerprint of NJFE metabolite

**FIGURE 7:**
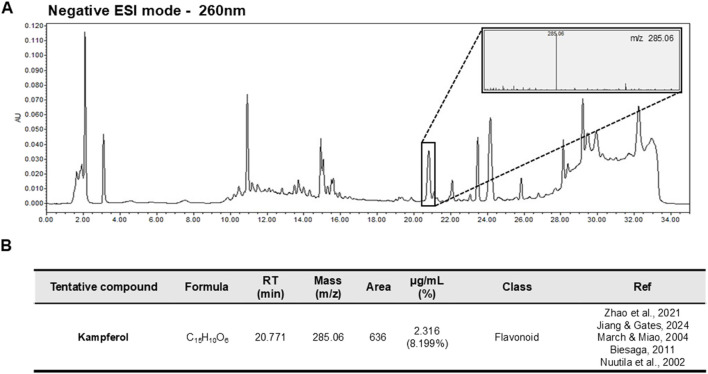
Representative LC-MS Chromatogram and Tentative Metabolite List of NJFE. **(A)** Representative LC-MS chromatogram of NJFE acquired under negative electrospray ionization (ESI-) mode. The peaks represent detected ions corresponding to various metabolites. The top right panel shows the parent ion pattern of a specific peak. **(B)** Table listing the M/Z value of the peak identified from LC-MS analysis of NJFE. The metabolite was putatively identified as kaempferol by comparing the observed M/Z value and parent ion pattern with literature reports and metabolite databases.

The presence of kaempferol derivatives in *Neoshirakia japonica* (Siebold & Zucc.) Esser [Euphorbiaceae] has been previously reported in the literature (Zhao et al., 2021). Kaempferol was tentatively identified in NJFE based on mass spectral data obtained in this study by comparison with an LC-MS spectral library and previously reported metabolites isolated from *Neoshirakia japonica* (Siebold & Zucc.) Esser [Euphorbiaceae] (Jiang and Gates, 2024; March and Miao, 2004; Zhao et al., 2021) (Figures 7A,B). Parent ion analysis using LC-MS revealed a prominent signal corresponding to the free form of kaempferol ([M-H]^-^, m/z 285.06), which differs from the kaempferol derivatives commonly reported in the literature. It has been documented that kaempferol glycosides can be hydrolyzed into their aglycone forms under certain extraction conditions or due to specific sample characteristics, though it is also possible that the free form originally existed in the extract (Biesaga, 2011; Nuutila et al., 2002). The concentration of kaempferol in NJFE (10 ppm) was determined to be 2.316 μg/mL. Kaempferol has been reported to exhibit anti-obesity and anti-inflammatory activities, supporting the relevance of our findings (Alam et al., 2020; Bian et al., 2022). These findings suggest that kaempferol may be one of the bioactive metabolites contributing to the observed anti-obesity and anti-inflammatory effects of NJFE. The chemical fingerprint established here provides a foundational understanding of the bioactive metabolites contributing to the anti-obesity and anti-inflammatory effects demonstrated by NJFE.

## 4 Discussion

*Neoshirakia japonica* (Siebold & Zucc.) Esser [Euphorbiaceae] has been traditionally used in ethnopharmacology for its efficacy in dispelling “wind-dampness” and “damp-heat,” conditions associated with pain, swelling, and metabolic imbalance. These traditional concepts are closely aligned with chronic inflammation (Ge et al., 2020) and metabolic disorders such as obesity (Zhao et al., 2023a) in modern medicine. We present the rationale and validity of our study based on the traditional use records of *Neoshirakia japonica* (Siebold & Zucc.) Esser [Euphorbiaceae], and this work represents the first study to reinterpret such traditional knowledge from a modern perspective by providing molecular mechanisms together with *in vivo* evidence. This study therefore carries medical significance, as it may directly contribute to the development of therapeutic strategies for human obesity and metabolic disorders.

We propose the following sequential mechanistic model to explain the anti-obesity and anti-inflammatory effects of NJFE in obesity.

1. Obesity is characterized by enhanced adipocyte differentiation and increased lipid accumulation. NJFE acts at the early stage of adipogenic differentiation to effectively inhibit adipocyte formation and block maturation, thereby ameliorating the obese phenotype in mouse models. Specifically, NJFE suppresses the expression of key transcription factors essential for adipocyte maturation, including PPARγ and C/EBPα, while upregulating anti-adipogenic regulators such as CHOP10 and GATA2 (Figures 1–4; Supplementary Figure S2) (Berger, 2005; Lee et al., 2019; Park et al., 2025). These molecular changes are consistent with significant reduction in adipocyte size observed in histological analysis, suggesting that the NJFE-induced downregulation of PPAR**γ** and C/EBP**α** effectively suppresses lipid accumulation and adipocyte hypertrophy (Figures 2A,B, 3E). This indicates that the anti-adipogenic effects observed at the gene expression level are faithfully reflected at the tissue level, supporting the notion that the anti-obesity effects of NJFE extend beyond transcriptional regulation to functional and morphological outcomes. By targeting the early phase of adipogenesis, NJFE effectively limits the overall increase in adipocyte number, suggesting its potential utility as an anti-obesity agent. Numerous studies have reported that natural metabolites exert anti-obesity effects by modulating adipogenesis-related pathways (Lyu et al., 2025; Ono et al., 2006; Widyananda et al., 2025). In addition, NJFE appears to enhance mitochondrial function in adipocytes, thereby increasing energy metabolism and expenditure, which further potentiates its anti-obesity efficacy (Supplementary Figure S3). This mechanism is particularly important, as mitochondrial dysfunction and metabolic inefficiency are commonly observed in obesity. Notably, increased mitochondrial activity is considered a key biomarker in evaluating the anti-obesity efficacy of functional metabolites (Luo et al., 2024).
2. Hypertrophic adipocytes in obesity secrete pro-inflammatory cytokines (e.g., TNFα, IL-6, MCP1) and free fatty acids, which act as chemotactic signals to recruit macrophages into adipose tissue (Xu et al., 2003). NJFE reduces the secretion of TNFα and IL-6 from hypertrophic adipocytes (Figure 6A), thereby suppressing macrophage infiltration into adipose tissue (Figure 3A).
3. Infiltrated macrophages within adipose tissue polarize toward the M1 phenotype via activation of the NF-κB signaling pathway and secrete TNFα, IL-6, MCP1, and iNOS, thereby promoting chronic inflammation (Tam et al., 2010). NJFE significantly attenuates not only macrophage-derived inflammatory responses but also the adipocyte inflammation induced by macrophage-conditioned media (Figures 3, 5, 6). Specifically, NJFE downregulates the NF-κB pathway and its downstream inflammatory mediators, including TNFα, IL-6, MCP1, and iNOS, in both macrophage-stimulated adipocyte models and in obese mice. Furthermore, NJFE restores the expression of adiponectin, which is typically reduced by inflammation, thereby improving metabolic and anti-inflammatory indicators (Liu and Li, 2025).
4. In obese state, adipose tissue functions not only as an energy reservoir but also as an immunologically active inflammatory organ. Pro-inflammatory cytokines produced by infiltrated macrophages disrupt insulin receptor signaling, contributing to systemic insulin resistance in adipose tissue, liver, and skeletal muscle (Bourlier and Bouloumié, 2009). This insulin resistance leads to impaired glucose homeostasis and ultimately induces glucose intolerance (Choi et al., 2005). NJFE effectively improves inflammation-induced glucose intolerance in obese mouse models (Figure 2). These findings suggest that NJFE alleviates the chronic low-grade inflammation commonly observed in obesity by disrupting the inflammatory crosstalk between macrophages and adipocytes. Therefore, NJFE may serve not only as an anti-obesity agent but also as a promising therapeutic candidate for the prevention and treatment of obesity-associated inflammatory complications.

We propose that the anti-obesity and anti-inflammatory effects of NJFE may be attributed to the bioactive metabolites present within the extract. Based on mass spectra obtained through LC-MS analysis and reference comparisons with metabolites previously reported in *Neoshirakia japonica* (Siebold & Zucc.) Esser [Euphorbiaceae], we identified kaempferol as a tentative metabolite in NJFE (Jiang and Gates, 2024; March and Miao, 2004; Zhao et al., 2021). Kaempferol is a well-known dietary flavonoid that has been reported to inhibit the differentiation of 3T3-L1 adipocytes and to protect mice from high-fat diet (HFD)-induced obesity by reducing lipid accumulation, improving glucose homeostasis (Bian et al., 2022; Torres-Villarreal et al., 2019). In addition, kaempferol is recognized for its potent anti-inflammatory activity. It suppresses the activation of Toll-like receptor 4 (TLR4), reduces the expression and secretion of pro-inflammatory cytokines, and inhibits NF-κB signaling (Alam et al., 2020). These findings align well with our hypothesis, indicating that the metabolic protective effects observed with NJFE treatment in obese mice may be, at least in part, attributable to the presence and activity of kaempferol. Green tea (Camellia sinensis) is a well-known natural product with established anti-obesity and anti-inflammatory effects (Huang et al., 2014; Park et al., 2012). Given that the kaempferol content in green tea has been reported to be 2.8114 μg/mL, the concentration of kaempferol detected in NJFE (2.316 μg/mL) represents a comparably meaningful level (Uba et al., 2022). This finding suggests that the anti-obesity and anti-inflammatory effects of NJFE are likely mediated by the presence of kaempferol. Furthermore, although NJFE is a complex extract containing various bioactive metabolites, this study focused on kaempferol, which showed relatively high abundance and clear analytical characteristics. This was a strategic choice to enhance the clarity and reproducibility of our results. However, we do not rule out the possibility that minor metabolites or synergistic interactions among metabolites may have contributed to the observed bioactivity. Therefore, future studies should aim to comprehensively identify and evaluate the biological functions of other metabolites in NJFE to elucidate their potential combined effects.

Orlistat, a well-established standard anti-obesity agent, is known to significantly reduce total cholesterol and LDL-C while modestly increasing HDL-C by inhibiting intestinal fat absorption (Hutton and Fergusson, 2004). In the present study, NJFE also improved total cholesterol, LDL-C, and HDL-C to a comparable extent. However, these effects appear to be mediated not by inhibition of intestinal lipid absorption, but rather through attenuation of adipose tissue inflammation and enhancement of adipocyte metabolic function. This mechanistic distinction suggests that NJFE may provide lipid profile benefits similar to those of Orlistat while potentially avoiding the gastrointestinal side effects commonly associated with fat absorption inhibitors. Thus, our findings highlight the advantages of a non-pharmacological, natural product–based approach, which may offer a favorable safety profile compared with conventional therapeutics.

Collectively, our findings demonstrate that NJFE is a promising natural material capable of alleviating obesity and its associated metabolic disorders. It improves metabolic health through multiple mechanisms, including the inhibition of adipogenesis, anti-inflammatory effects, and the enhancement of mitochondrial function, ultimately mitigating complications associated with obesity. This multi-targeted mode of action suggests that NJFE may serve as a novel therapeutic strategy to overcome the limitations of current anti-obesity drugs that rely on single-target approaches.

Nevertheless, this study has several limitations that warrant further investigation. The current findings are based on animal and cell models, which limit the generalizability of our results to human physiology. Therefore, future studies should incorporate human-derived adipocytes or three-dimensional tissue models to validate the medicinal potential of NJFE. In addition, considering the potential of NJFE as a functional food or therapeutic agent, comprehensive evaluations of its pharmacokinetics (including its absorption, metabolism, distribution, and excretion), safety, and efficacy are essential. Long-term studies are particularly important for assessing its sustained effects and potential toxicity. Moreover, the applicability of NJFE to other metabolic disorders, such as fatty liver disease and type 2 diabetes, should also be explored to expand its therapeutic scope.

## Data Availability

The datasets presented in this study can be found in online repositories. The names of the repository/repositories and accession number(s) can be found in the article/Supplementary Material.

## Abbreviations

NJFE, *Neoshirakia japonica* (Siebold & Zucc.) Esser [Euphorbiaceae] fruit extract; HFD, High-fat diet; eWAT, Epididymal white adipose tissue; iWAT, Inguinal white adipose tissue; rWAT, Retroperitoneal white adipose tissue; T-CHO, Total cholesterol; LDL-C, Low-density lipoprotein cholesterol; HDL-C, High-density lipoprotein cholesterol; TNFα, Tumor Necrosis Factor alpha; PPARγ, Peroxisome Proliferator-Activated Receptor gamma; C/EBPα, CCAAT/Enhancer-Binding Protein alpha; Ap2, Adipocyte Protein 2; Adipoq, Adiponectin; HSL, Hormone-Sensitive Lipase; LPL, Lipoprotein Lipase; IL-6, Interleukin-6; iNOS, Inducible Nitric Oxide Synthase; COX2, Cyclooxygenase-2; MCP1, Monocyte Chemoattractant Protein-1; CPT1, Carnitine Palmitoyltransferase 1; NF-κB, Nuclear Factor kappa-light-chain-enhancer of activated B cells; IRS1, Insulin receptor substrate 1; IRβ, Insulin receptor β.
