# Supplementary material for: Neoshirakia japonica (Siebold & Zucc.) Esser [Euphorbiaceae] fruit suppresses obesity and obesity-induced inflammation in adipocytes, macrophages, and obese mice
Source: Front Pharmacol. 2025 Sep 29;16:1647343. doi: 10.3389/fphar.2025.1647343 (PMC12515811; doi:10.3389/fphar.2025.1647343)
Supplement: Supplementary file 1 [file DataSheet1.pdf]

[Supplymentary Materials]

Neoshirakia japonica (Siebold & Zucc.) fruit suppresses obesity and obesity-induced inflammation in adipocytes, macrophages, Sand obese mice

Eunbi Lee[1,2], Juhye Park[1,2], Ju-Ock Nam[1,2,3,4,*]

[1]Department of Food Science and Biotechnology, Kyungpook National University, Daegu 41566, Republic of Korea.
[2]Department of Integrative Biology, Kyungpook National University, Daegu 41566, Republic of Korea
[3]Research Institute of Tailored Food Technology, Kyungpook National University, Daegu 41566, Republic of Korea
[4]Department of Advanced Bioconvergence, Kyungpook National University, Daegu 41566, Republic of Korea

A

g	ND	HFD	10	20
BAT	0.051 ± 0.0137	0.082 ± 0.0123	0.079 ± 0.0137	0.072 ± 0.0130
Liver	1.005 ± 0.1032	1.114 ± 0.1976	1.082 ± 0.1497	1.028 ± 0.1045
Spleen	0.061 ± 0.057	0.073 ± 0.0115	0.077 ± 0.0236	0.074 ± 0.0167
Kidney	0.309 ± 0.0301	0.306 ± 0.0306	0.314 ± 0.0306	0.311 ± 0.0294

B

	ND	HFD	10	20
GOT(U/L)	117.2 ± 31.72	182.2 ± 64.18	143.6 ± 37.23	164.3 ± 29.01
GPT(U/L)	45.8 ± 21.53 #	99.0 ± 29.02	74.3 ± 14.97	48.5 ± 4.65 *
BUN(mg/dL)	33.0 ± 5.75 #	19.9 ± 1.44	19.5 ± 2.36	19.3 ± 3.71
CREA(mg/dL)	0.6 ± 0.02 #	0.7 ± 0.06	0.6 ± 0.02 *	0.6 ± 0.03 *

C

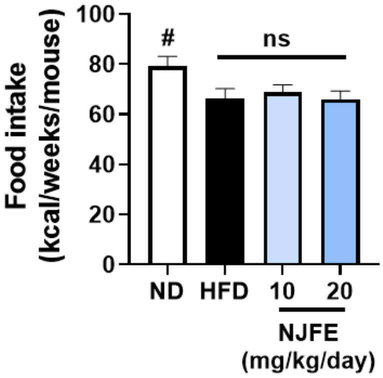

Figure S1. NJFE does not cause toxicity in the liver, kidneys, or spleen when administered to high-fat diet (HFD)-induced obese mice. (A) A table showing the weights of BAT, liver, spleen, and kidney in mice. The tissue weights are presented in grams (g) and expressed as mean ± standard deviation. (B) A table showing liver and kidney toxicity markers in mouse serum. It presents four indicators: GOT and GPT (liver toxicity) and BUN and CREA (kidney toxicity). '#' indicates a statistically significant difference between control groups (p < 0.05 when comparing ND and HFD). '*' indicates a p < 0.05 compared to HFD. '**' indicates a p < 0.01 compared to line HFD. (C) A graph showing the food intake of mice. The weight of the consumed food was converted to kilocalories (kcal) and calculated by dividing by the number of experimental days. '#' indicates a statistically significant difference between control groups (p < 0.05 when comparing ND and HFD).

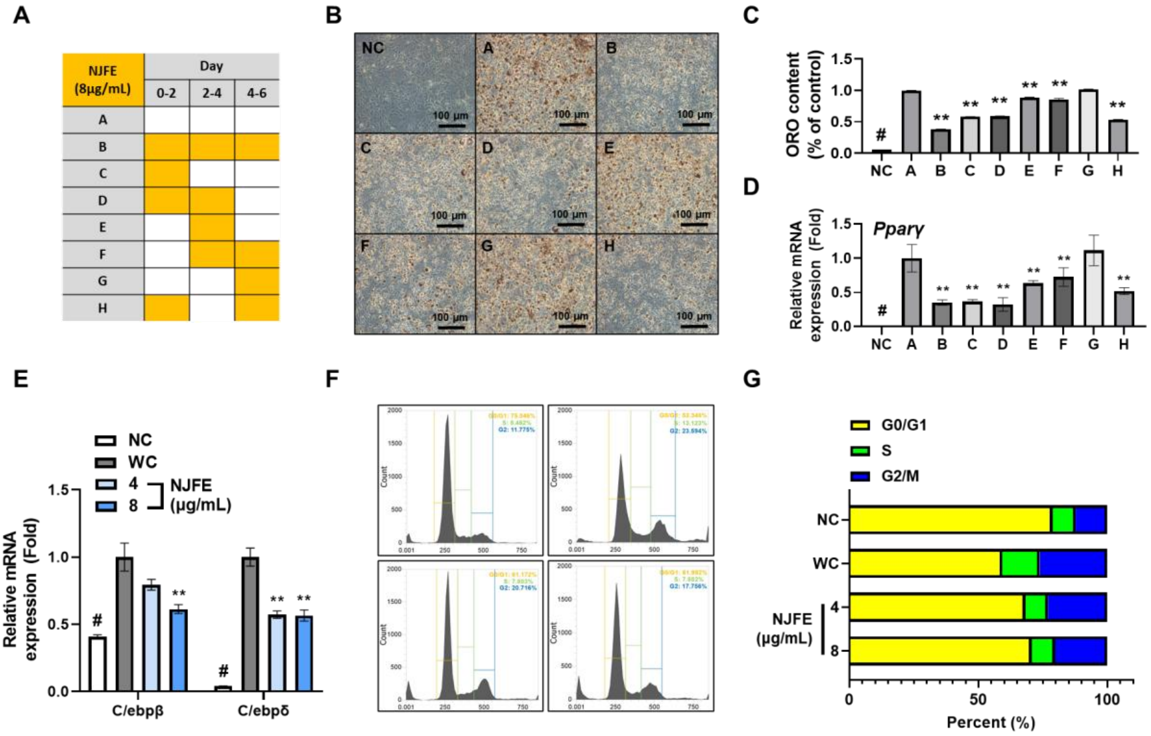

Figure S2. The inhibitory effect of NJFE on the early stage of adipocyte differentiation. (A) Schematic representation of the experimental design. 3T3-L1 preadipocytes were treated with NJFE (8 μg/mL) at different time points during adipocyte differentiation (days 0-2, 2-4, or 4-6) according to the indicated groups (A-H). (B) Representative Oil Red O staining images of differentiated adipocytes treated with SJF at different stages of differentiation. Images were taken at 200× magnification. The scale bar represents 100 μm. (C) Quantification of Oil Red O (ORO) staining. The absorbance of ORO contents from stained cells was measured at 450 nm to assess lipid accumulation. (D) Changes in *Pparγ* mRNA expression according to experimental design. '#' indicates a statistically significant difference between control groups ($p < 0.05$ when comparing NC and A). '**' indicates a $p < 0.01$ compared to A. (E) mRNA expression levels of C/ebpβ and C/ebpδ in 3T3-L1 preadipocytes after 16 hours of differentiation induction, during which NJFE was co-treated. '**' indicates a $p < 0.01$ compared to WC. '#' indicates a statistically significant difference between control groups ($p < 0.05$ when comparing NC and WC). (F) Flow cytometric analysis of the cell cycle in PI-stained 3T3-L1 adipocytes. (G) Quantitative analysis of cell cycle distribution.

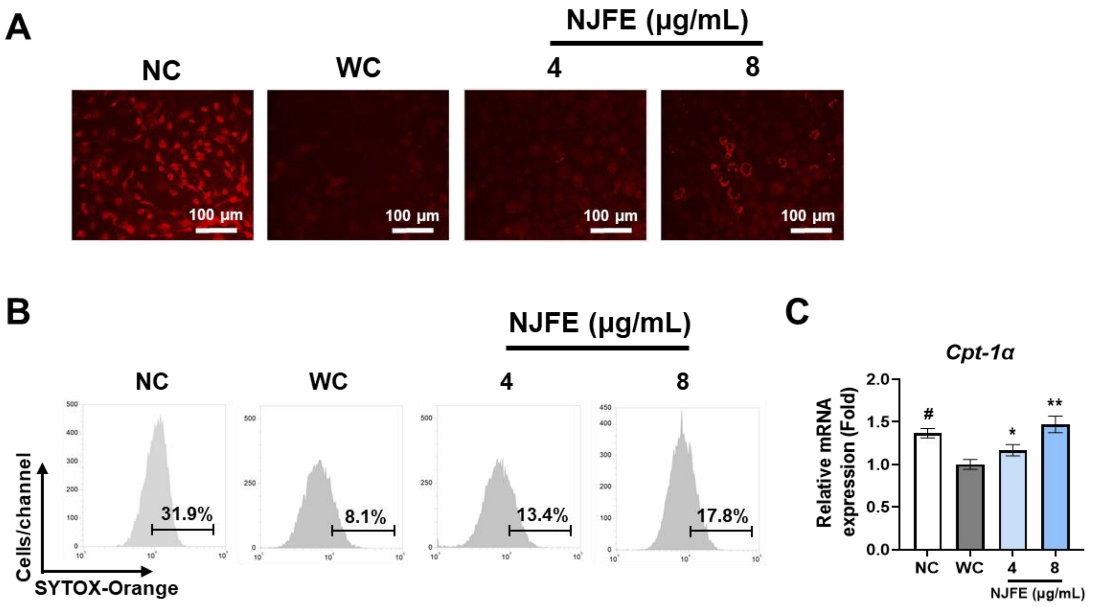

Figure S3. NJFE improves mitochondrial function in 3T3-L1 adipocytes. (A) Mitotracker staining observed by fluorescence microscopy in 3T3-L1 adipocytes. Images were taken at 200× magnification. The scale bar represents 100 μm. (B) Mitotracker staining observed by flow cytometry in 3T3-L1 adipocytes. 1 x 104 cells were counted per group. (C) Relative mRNA expression levels of *Cpt-1α.* RT-qPCR result was normalized to β-actin. The result of PCR is presented as fold changes relative to the expression levels in the control (WC) group. All data are presented as mean ± standard deviation. '#' indicates a statistically significant difference between control groups ($p < 0.05$ when comparing NC and WC). '*' indicates a $p < 0.05$ compared to WC. '**' indicates a $p < 0.01$ compared to WC.

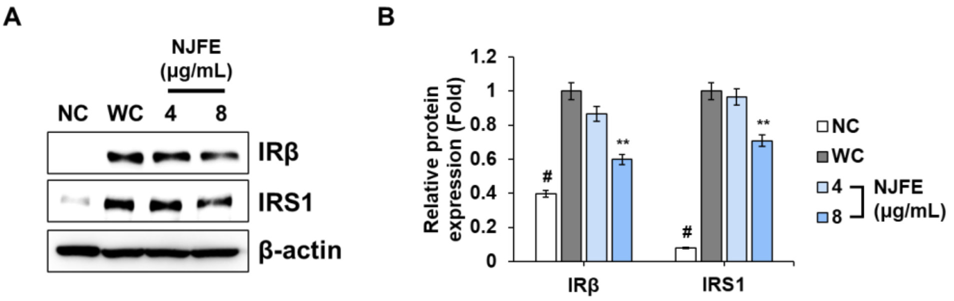

Figure S4. The effect of NJFE on the expression of IR and IRS1 during adipocyte differentiation. 3T3-L1 preadipocytes were induced to differentiate in the presence of NJFE for 8 days. (A) Representative western blot bands showing the protein expression levels of IRβ and IRS1 in cells treated with different concentration of NJFE (4, 8 μg/mL). (B) A graph of IRβ and IRS1 protein expression levels quantified based on western blot band intensities. All data are presented as mean ± standard deviation. '#' indicates a statistically significant difference between control groups ($p < 0.05$ when comparing NC and WC). '**' indicates a $p < 0.01$ compared to WC.

Table S1. Custom-designed RT-qPCR primers used in the experiment

Genes	Sequence	
F4/80	Forward	GTATTCCAACTGCTCTAA
	Reverse	CATTCATCTTCATCTTCAC
Tnfα	Forward	TGGAACTGGCAGAAGAGGCACT
	Reverse	AGAGGCTGAGACATAGGCACCG
Pparγ	Forward	TTTTCAAGGGTGCCAGTTTC
	Reverse	AATCCTTGGCCCTCTGAGAT
C/ebpα	Forward	TTACAACAGGCCAGGTTTCC
	Reverse	GGCTGGCGACATACAGATCA
Chop10	Forward	GTGGGTAGCTTGGCTGACA
	Reverse	GTTTCGGGAGCGAGAGGT
Gata2	Forward	GCAGAGAAGCAAGGCTCGC
	Reverse	CGGCCCTCACACAGTTGAC
Ap2	Forward	TGGACGTTGTCTAGGGGGTA
	Reverse	AAGGTGAAGAGCATAACCCT-
Adipoq	Forward	ACCTACGACCAGTATCAGGAAAAG
	Reverse	ACTAAGCTGAAAGTGTGTCGACTG
Hsl	Forward	CAGAAGGCACTAGGCGATG
	Reverse	GGGCTTGCGTCCACTTAGTTTC
Lpl	Forward	CTGGTGGGAAATGATGTGG
	Reverse	TGGACGTTGTCTAGGGGGTA
C/ebpβ	Forward	AAGAAGACGGTGGACAAGCTG
	Reverse	TGCTCCACCTTCTTCTGCAGC
C/ebpδ	Forward	TCCACGACTCCTGCCATGTAC
	Reverse	AAGAGTTCGTCGTGGCACAG
β-actin	Forward	CGTGCGTGACATCAAAGAGAA
	Reverse	GCTCGTTGCCAATAGTGATGA

Table S2. Western blot antibodies used in the experiments

Genes	Company	Product No.	Dilution
**PPARγ**	Santa Cruz Biotechnology	SC-7273	1:500
**C/EBPα**	Cell Signaling Technology	2295S	1:1000
**p-NF-κB**	Cell Signaling Technology	8242S	1:1000
**NF-κB**	Cell Signaling Technology	3033S	1:1000
**Adiponectin**	Abcam	AB22554	1:1000
**iNOS**	Cell Signaling Technology	13120S	1:1000
**COX2**	Cell Signaling Technology	12282S	1:1000
**β-actin**	Santa Cruz Biotechnology	SC-47778	1:500
